# Supplementary figures and images for: Genome-Wide Association Study Reveals Candidate Genes for Flowering Time in Cowpea (Vigna unguiculata [L.] Walp.)
Source: Front Genet. 2021 Jun 16;12:667038. doi: 10.3389/fgene.2021.667038 (PMC8242349; doi:10.3389/fgene.2021.667038)

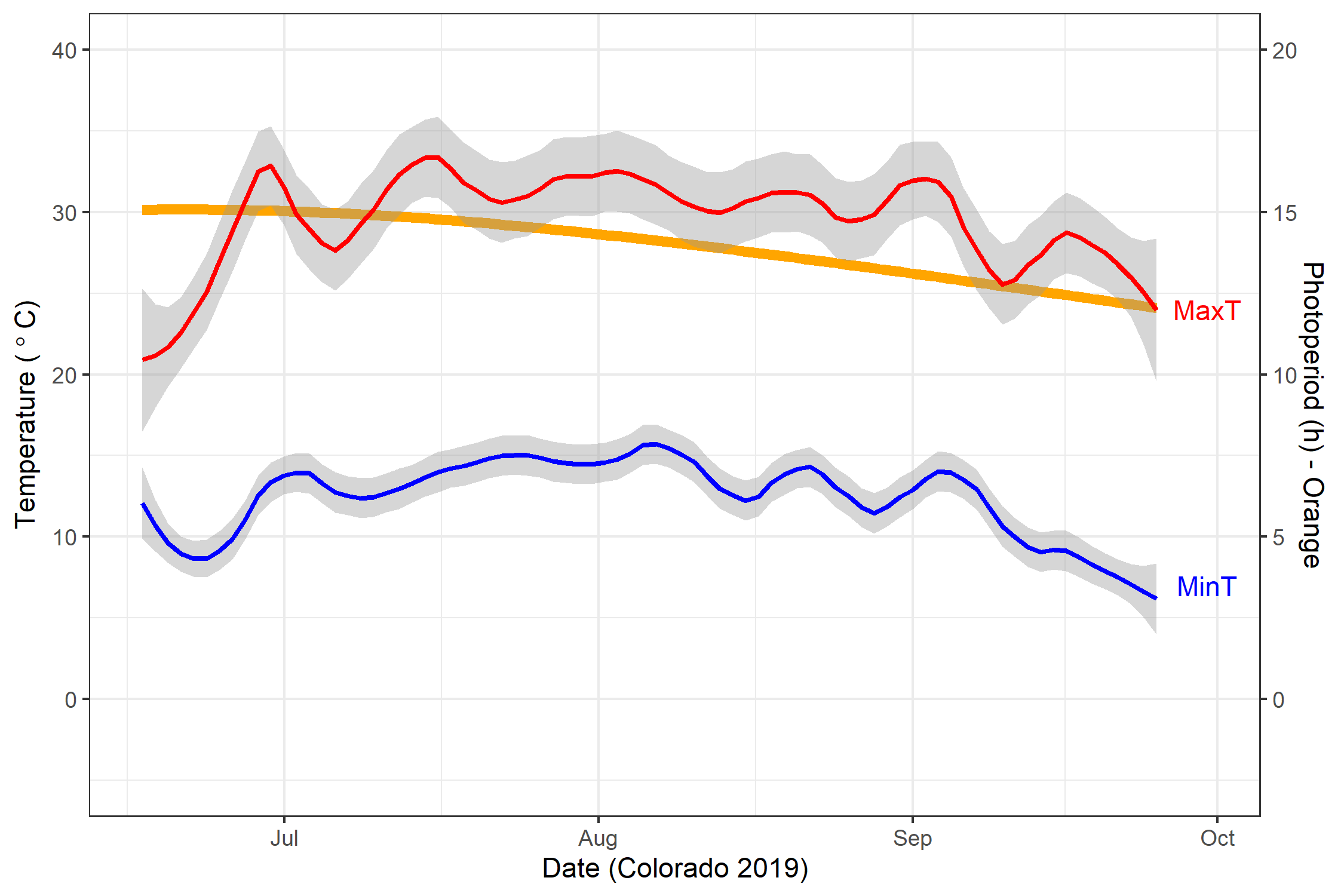

Supplement: Supplementary Figure 1 — Daily maximum (MaxT) and minimum (MinT) temperature and photoperiod (orange line) in Ft. Collins, CO during the trial in 2019. [file Image_1.TIFF]

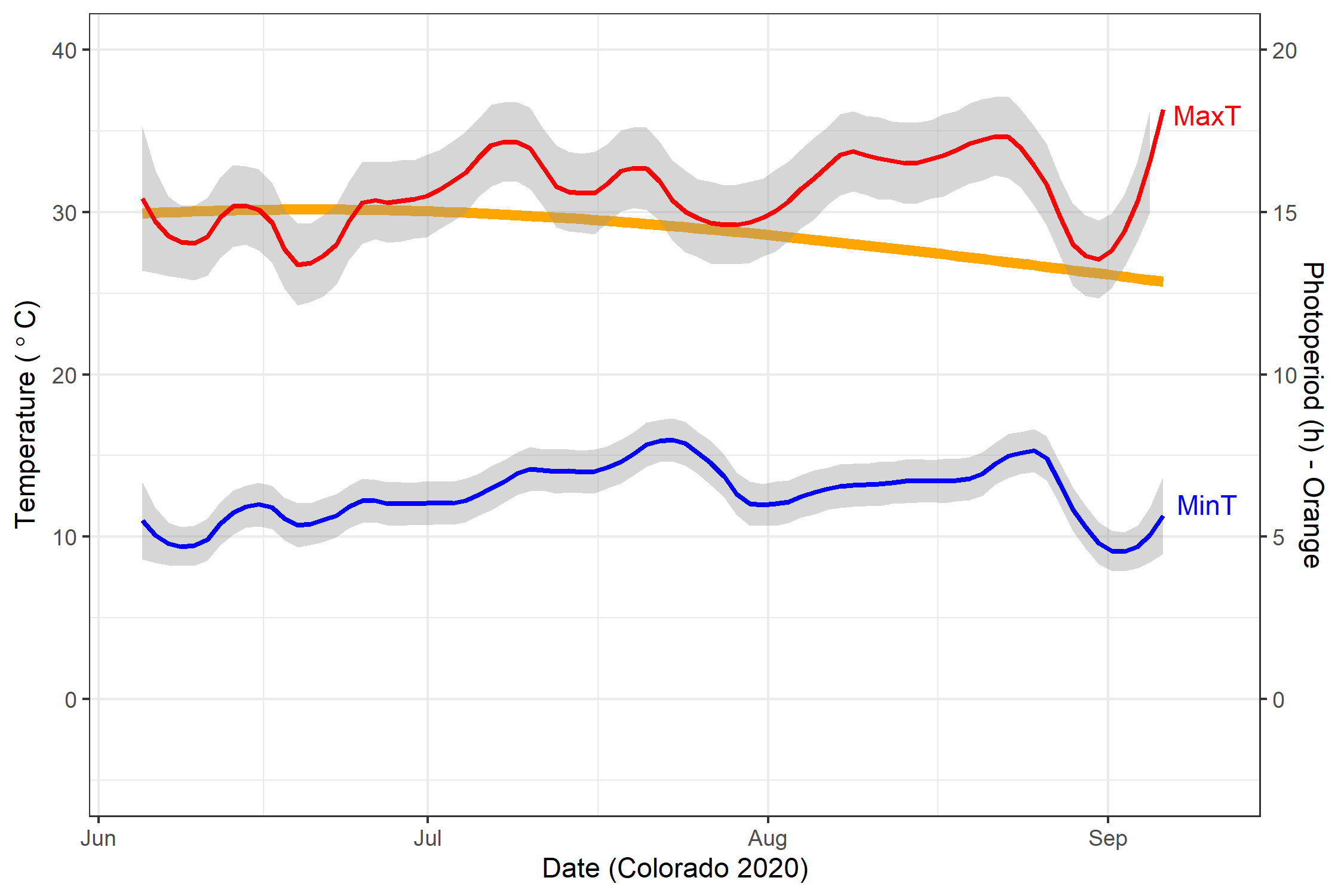

Supplement: Supplementary Figure 2 — Daily maximum (MaxT) and minimum (MinT) temperature and photoperiod (orange line) in Ft. Collins, CO during the trial in 2020. [file Image_2.TIFF]

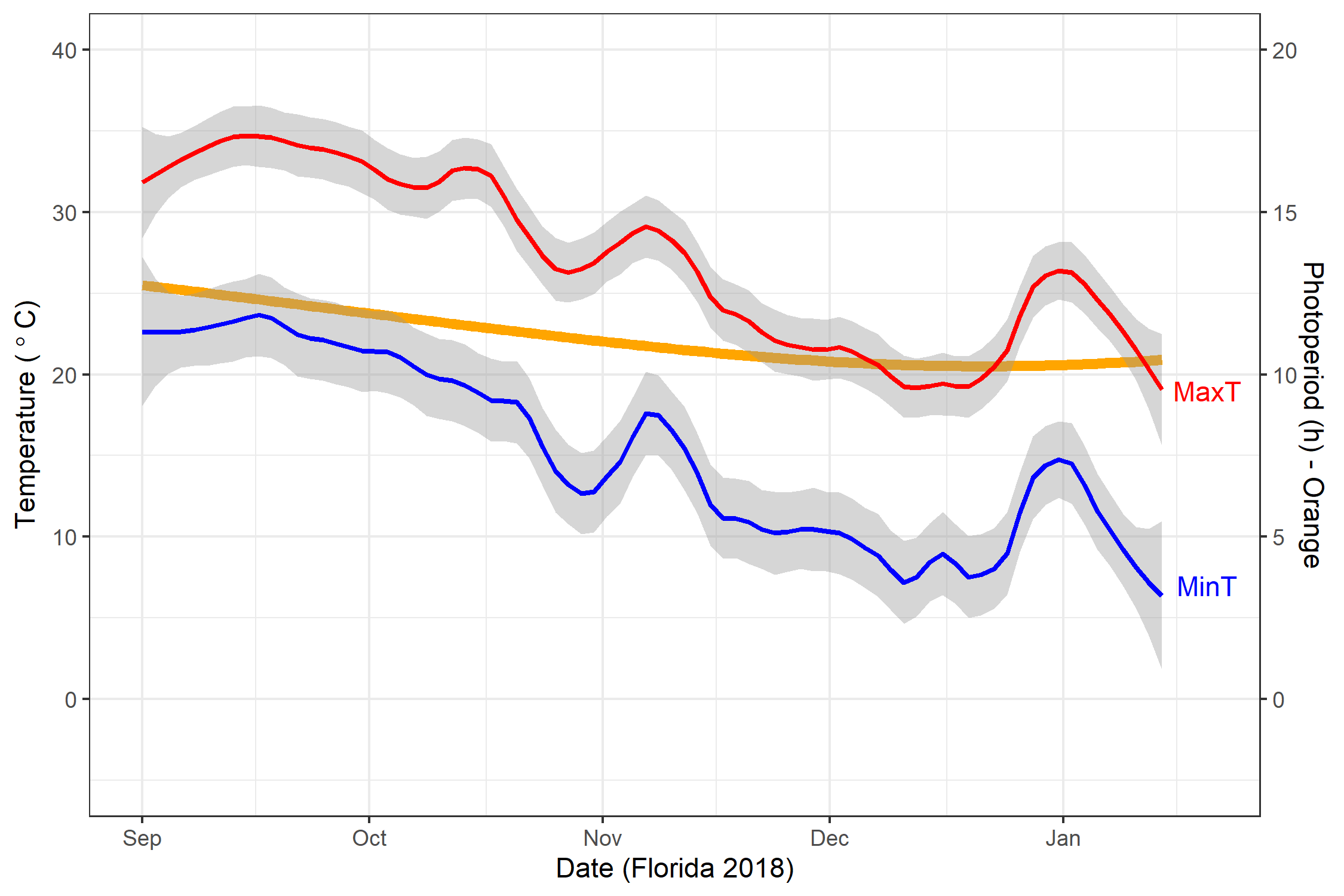

Supplement: Supplementary Figure 3 — Daily maximum (MaxT) and minimum (MinT) temperature and photoperiod (orange line) in Citra, FL during the trial in 2018. [file Image_3.TIFF]

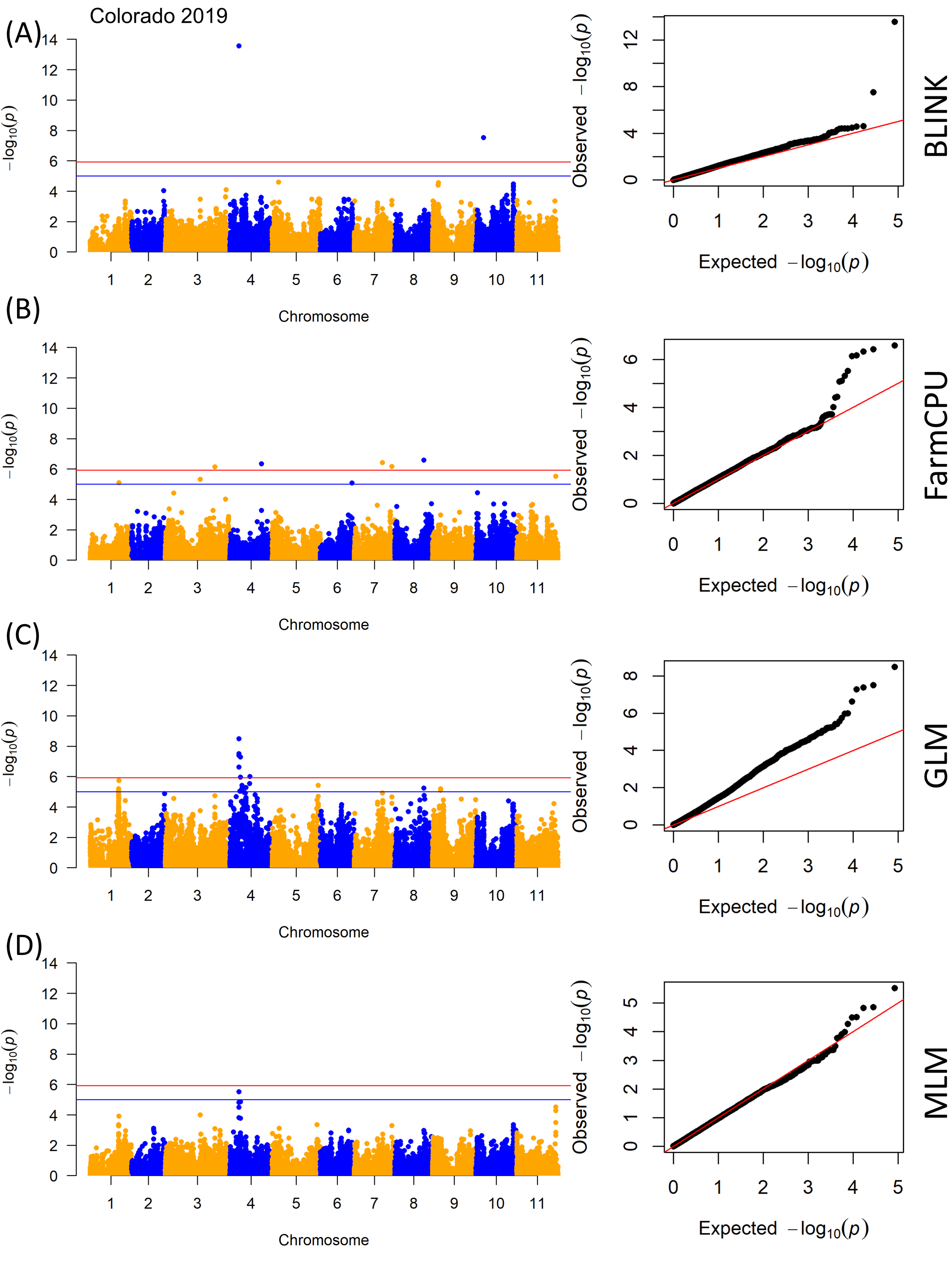

Supplement: Supplementary Figure 4 — Manhattan plots from the GWAS analysis pertaining to 368 accessions of the cowpea mini-core collection planted in 2019 in Colorado. Left panel: Negative log10-transformed p-values for each SNP (y axis) are plotted against the chromosomal position (y axis). The red line represents Bonferroni-corrected threshold of 0.05 for genome-wide statistically significant associations and the blue line shows suggestive associations (p = 1 × 10^–5^). Right panel shows the QQ plots where x-axis is expected negative log p-values and the y-axis is observed negative log p-values. GWAS results for days to flowering using (A) BLINK; (B) FarmCPU; (C) GLM; and (D) MLM. [file Image_4.TIFF]

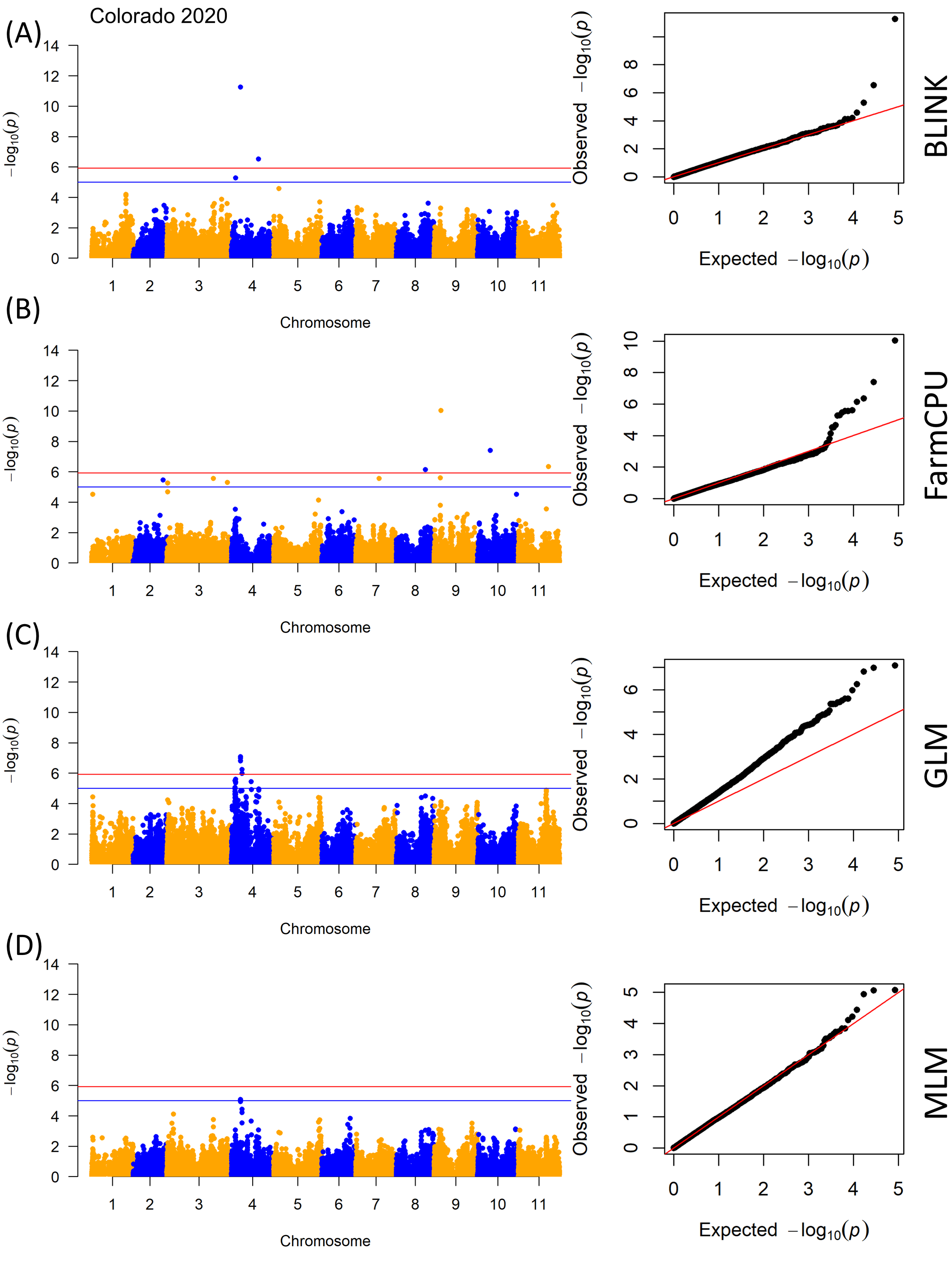

Supplement: Supplementary Figure 5 — Manhattan plots from the GWAS analysis pertaining to 368 accessions of the cowpea mini-core collection planted in 2020 in Colorado. Left panel: Negative log10-transformed p-values for each SNP (y axis) are plotted against the chromosomal position (y axis). The red line represents Bonferroni-corrected threshold of 0.05 for genome-wide statistically significant associations and the blue line shows suggestive associations (p = 1 × 10^–5^). Right panel shows the QQ plots where x-axis is expected negative log p-values and the y-axis is observed negative log p-values. GWAS results for days to flowering using (A) BLINK; (B) FarmCPU; (C) GLM; and (D) MLM. [file Image_5.TIFF]

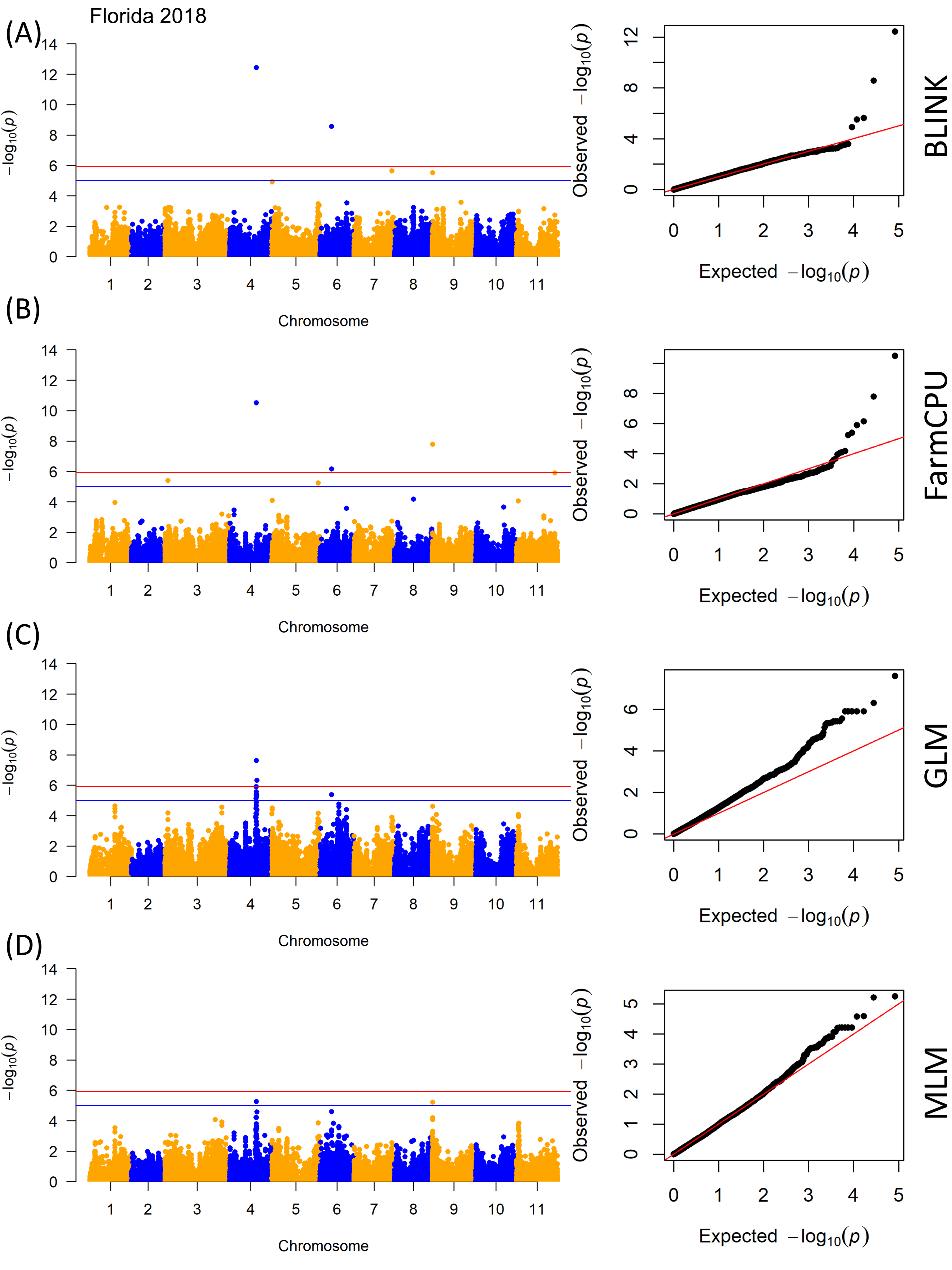

Supplement: Supplementary Figure 6 — Manhattan plots from the GWAS analysis pertaining to 292 accessions of the cowpea mini-core collection planted in 2018 in Florida. Left panel: Negative log10-transformed p-values for each SNP (y axis) are plotted against the chromosomal position (y axis). The red line represents Bonferroni-corrected threshold of 0.05 for genome-wide statistically significant associations and the blue line shows suggestive associations (p = 1 × 10^–5^). Right panel shows the QQ plots where x-axis is expected negative log p-values and the y-axis is observed negative log p-values. GWAS results for days to flowering using (A) BLINK; (B) FarmCPU; (C) GLM; and (D) MLM. [file Image_6.TIFF]

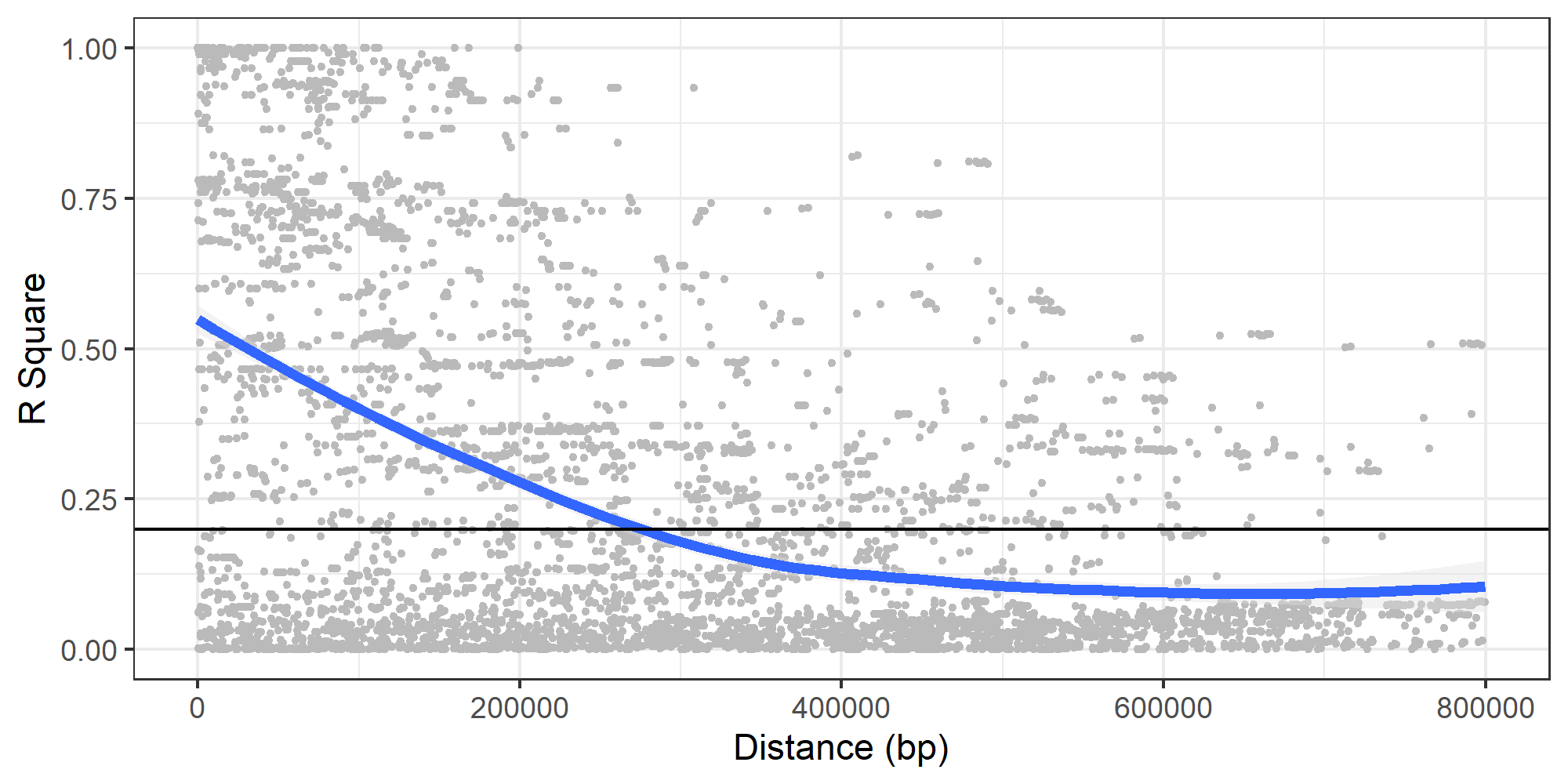

Supplement: Supplementary Figure 7 — Linkage disequilibrium (LD) decay plot of the 368 accessions of the UCR mini-core collection. [file Image_7.PNG]
